# Dietary amino acid patterns and cardiometabolic risk factors among subjects with obesity; a cross-sectional study

**DOI:** 10.1186/s12902-024-01549-w

**Published:** 2024-02-14

**Authors:** Fatemeh Abdi, Milad Mohammadzadeh, Mahdieh Abbasalizad-Farhangi

**Affiliations:** 1 Department of Community Nutrition, Faculty of Nutrition, Tabriz University of Medical Sciences, Attar Neyshabouri, Daneshgah Blv, Tabriz, Iran; 2 Department of Clinical Nutrition and Dietetics, Faculty of Nutrition Sciences and Food Technology, Shahid Beheshti University of Medical Sciences, Tehran, Iran

**Keywords:** Amino acids, Dietary protein, Factor analysis, Cardi-metabolic factors, Obesity, Amino acid pattern, Cardio-metabolic risk factors, Diet

## Abstract

**Background:**

The prevalence of obesity is a growing global public health concern. Certain dietary amino acids have been shown to have a potential therapeutic role in improving metabolic syndrome parameters and body composition in individuals with obesity. However, some amino acids have been linked to an increased risk of cardiometabolic disorders. This cross-sectional study aims to investigate the association between dietary amino acid patterns and cardiometabolic risk factors in individuals with obesity.

**Methods:**

This cross-sectional study included 335 participants with obesity (57.9% males and 41.5% females) from Tabriz and Tehran, Iran. The participants were between the ages of 20–50, with a body mass index (BMI) of 30 kg/m2 or higher, and free from certain medical conditions. The study examined participants’ general characteristics, conducted anthropometric assessments, dietary assessments, and biochemical assessments. The study also used principal component analysis to identify amino acid intake patterns and determined the association between these patterns and cardiometabolic risk factors in individuals with obesity.

**Results:**

Upon adjusting for potential confounders, the study found that individuals in the third tertiles of pattern 1 and 2 were more likely to have lower LDL levels (OR = 0.99 and 95% CI (0.98–0.99)) for both. Additionally, a significant decrease in total cholesterol was observed in the third tertiles of pattern 2 in model II (OR = 0.99, 95% CI (0.98–0.99)). These findings suggest a potential cardioprotective effect of these amino acid patterns in managing cardiometabolic risk factors in individuals with obesity.

**Conclusions:**

This study found that two identified amino acid patterns were associated with lower serum LDL and total cholesterol levels, while a third pattern was associated with higher serum triglycerides. The specific amino acids contributing to these patterns highlight the importance of targeted dietary interventions in managing cardiometabolic risk factors in individuals with obesity.

## Introduction

The escalating global prevalence of obesity, characterized by a body mass index (BMI) of 30 kg/m2 or higher, has become a major public health concern worldwide [1–3]. The World Health Organization (WHO) reports that the number of individuals affected by obesity has nearly tripled since 1975 [4]. In Iran, Tabrizi et al. found that approximately 25% of adults are obese [5]. Obesity is strongly associated with various cardio-metabolic disorders, including high blood pressure, elevated blood glucose, insulin resistance, high cholesterol, coronary heart disease, stroke, and certain types of cancers [6, 7]. Considering the high prevalence of obesity and its associated health consequences, achieving a healthy weight is of utmost importance. The pathophysiology of obesity is complex and multifactorial, involving both unmodifiable factors such as genetics, as well as modifiable factors such as sedentary lifestyle and diet [8, 9].

Emerging research suggests that specific dietary amino acids may have a potential therapeutic role in improving metabolic syndrome parameters and body composition in individuals with obesity [10]. Epidemiological studies have also explored the relationship between dietary amino acid patterns and various chronic diseases, including hypertension, type 2 diabetes, and metabolic biomarkers [11–13]. Certain dietary amino acids have been linked to an increased risk of hypertension and vascular dysfunction [14–16]. For example, amino acids like tyrosine and tryptophan have been shown to induce a vasodilator response in specific regions of the brain or blood vessel walls [15, 16]. Teymoori et al. discovered that a high intake of branched-chain amino acids (BCAA), alcoholic amino acids, and proline may increase the risk of developing hypertension [13]. Conversely, plant-derived L-arginine has been associated with a lower risk of cardiovascular disease (CVD) events due to its ability to produce nitric oxide [17]. A randomized controlled trial conducted by Mone et al. to evaluated the effect of L-arginine on cardiac rehabilitation revealing that L-arginine enhances the response to cardiac rehabilitation regardless of factors such as age, gender, baseline functional capacity, and comorbid conditions [18]. Additionally, a case-control study suggested that a high consumption of BCAA, aromatic, and sulfuric amino acids may increase the likelihood of developing non-alcoholic fatty liver disease (NAFLD) [19]. NAFLD is associated with dyslipidemia and CVD [20]. Yu et al. also reported that higher intakes of BCAAs can elevate serum levels of specific lipids and increase the risk of dyslipidemia [21]. Furthermore, some studies have demonstrated novel roles for specific amino acids in the atherogenicity of macrophages through the modulation of triglyceride metabolism [22].

Despite the growing body of evidence regarding the relationship between dietary amino acids and chronic diseases, there is a lack of cross-sectional studies investigating the association between dietary amino acid patterns and the likelihood of cardiometabolic risk factors such as dyslipidemia, hyperglycemia, and hypertension in individuals with obesity. Therefore, the objective of this cross-sectional study is to determine whether dietary amino acid patterns can contribute to the alteration of cardiometabolic risk factors among individuals with obesity.

## Methods and materials

### Participants

This cross-sectional study included 335 individuals with obesity (57.9% males and 41.5% females) who were selected from two previous studies [23, 24] conducted in Tabriz and Tehran, Iran. Public announcements were made to invite eligible participants. When determining the sample size, we considered the relationship between dietary quality indices and obesity as an important dependent variable. We used G-power software with specific parameters, including a correlation coefficient (r) of 0.25, a significance level (α) of 0.05, and a desired statistical power of 80%. Based on these factors, the minimum sample size was estimated to be 150 individuals. To conduct a sex-stratified analysis and considering an 11% drop-out rate, the final sample size for the study was increased to 335 participants [25]. The study enrolled subjects between the age of 20–50 and a BMI of 30 kg/m2 and above. Pregnant, lactating, and menopausal women were excluded from the study. Other exclusion criteria included recent bariatric surgery, CVD, diabetes mellitus, hepatic and renal diseases, cancer, malabsorptive disorders, and the use of weight-altering drugs or supplements. Ethical considerations were taken into account, and written informed consent was obtained from all participants prior to their involvement in the study. The study concept was approved by the Ethics Committee of Tabriz University of Medical Sciences, Tabriz, Iran.

### General characteristics and anthropometric assessments

A face-to-face interview was conducted with each participant to gather sociodemographic information including sex, age, smoking status, education attainment, marital status, occupation, medical histories, and family size using a demographic questionnaire. The socio-economic status (SES) score was determined by considering Individual factors such as education level (highest level of educational attainment), family size, occupational status, and home ownership. The physical activity level of the participants was assessed using a concise form of the International Physical Activity Questionnaire (IPAQ) [26]. Body composition evaluations were performed using the bioelectrical impedance analysis (BIA) method (Tanita, BC-418 MA, Tokyo, Japan). Measurements of body fat percentage, fat mass (FM), fat free mass (FFM) and predicted muscle mass were obtained. The participants’ weights and heights were measured using a Seca scale (Seca co., Hamburg, Germany) and a wall-mounted stadiometer, respectively, and the results were rounded to the nearest 0.1 kg and 0.5 cm, respectively. Each subject’s waist circumference (WC) and hip circumference (HC) were measured to the nearest 0.1 cm using a fixed tension tape. WC was measured at the midpoint between the lower costal margin and the iliac crest, and HC was measured at the widest part of the hip. BMI and waist-to-hip ratio (WHR) were then calculated. Blood pressure was assessed twice in the same arm using a standard mercury sphygmomanometer, with a minimum rest period of 15 min between measurements. The average of the two readings was used for analysis. The participants appetite state was assessed using the visual analogue scale (VAS) [27] in the fasting state in the morning. The VAS questionnaire asked about hunger, satiety, fullness, and future food intake as well as cravings for sweet, salty, and fatty foods. The appetite was determined by the distance between the left side of the line and the mark.

### Dietary assessments

We used a validated semi-quantitative Food Frequency Questionnaire (FFQ) consisting of 168-item, adapted for Iranian population to assess dietary intakes of the subjects during the prior year [28]. The FFQ included a comprehensive list of food items that are frequently consumed in specified portion sizes in Iran. Participants were instructed to report the frequency and amount of each food item consumed, indicating whether they consumed them on a daily, weekly, monthly, or yearly basis during the past year. Household measures were then used to convert reported frequency of consumed foods and portion sizes for each food items into grams [29]. Due to the incompleteness of the Iranian Food Composition Table (FCT), the US Department of Agriculture (USDA) was utilized to analyze the energy and nutrient content of the reported foods. The intake of 18 individual amino acids from each food item was derived from the USDA. Amino acid intake was calculated as the frequency of consumption of each food item multiplied by the amino acid content of the food.

### Biochemical assessment

Following a 12-h fasting period, a morning venous blood sample of 10 ml was obtained from each participant and centrifuged at 4500 rpm for 10 min at 4°C to separate serum and plasma. The samples were then stored at -80°C until measurement. Commercial kits (Pars Azmoon, Tehran, Iran) were used to assess serum total cholesterol (TC), triglyceride (TG), high-density lipoprotein cholesterol (HDL-C), and fasting blood sugar (FBS). Furthermore, low-density lipoprotein cholesterol (LDL-C) level was evaluated by the Friedewald equation [30]. Serum insulin concentration was measured using enzyme-linked immunosorbent assay (ELISA) kits (Bioassay Technology Laboratory, Shanghai Korean Biotech, Shanghai City, China).

### Statistical analysis

Data analysis was carried out by SPSS version 27 (Statistical Package for Social Analysis, version 27, SPSS Inc., Chicago, IL, USA) with a significance level set at < 0.05. The principal component analysis (PCA) was used to determine amino acid intake patterns based on dietary intakes of amino acids (g/day). The ProMax rotation (an oblique rotation) was utilized due to the high correlation between the amino acids. Three patterns for amino acid intake were derived based on eigenvalues > 0.3, the scree plot, and the interpretability of the factors. Amino acid with an absolute component loading ≥ 0.40 were selected to describe each pattern. However, all amino acids contributed to the pattern score calculation. The Kaiser-Mayer-Olkin statistic, which serves as a measure of sampling adequacy, was 0.914, indicating good proportionality of factor analysis. We used Bartlett’s test of sphericity to assess the appropriateness of the correlation matrix for factor analysis and the *P* value was found to be < 0.001. Participants’ factor scores were calculated by multiplying the amino acid intake by their respective factor loadings in each amino acid pattern. All subjects were classified into tertiles based on the amino acid patterns. The normality distribution of the data was assessed through the Kolmogorov–Smirnov test. The participants’ baseline characteristics were presented as either median (interquartile range) and mean (standard deviation) for quantities variables, while frequency (%) were used for categorical variables. Chi-square test was used to compare the differences in categorical variables across different tertiles of amino acid patterns. For continuous variables, both the one-way analysis of variance (ANOVA) and the Kruskal-Wallis test were employed for parametric and non-parametric variables, respectively. Multivariate multinomial logistic regression with adjusted models (Model I: crude, Model II: adjusted for age and sex, Model III: adjusted for age, BMI, sex, physical activity, SES and energy intake) was used to obtain the odds ratio (OR) and 95% confidence interval (CI) for cardiometabolic risks across tertiles of amino acid patterns.

## Results

**Fig. 1:**
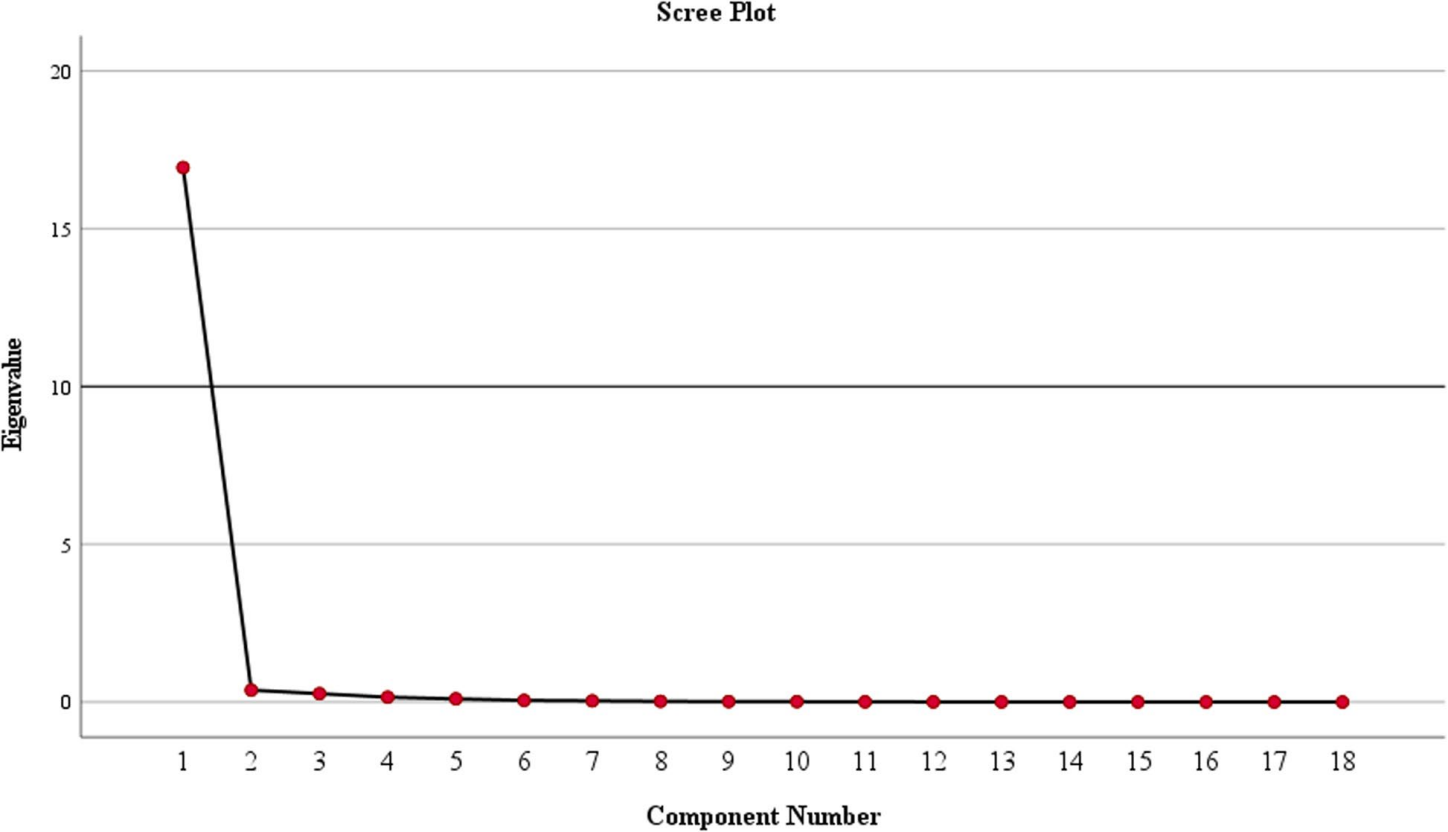
Scree plot representing the eigenvalues of dietary pattern analysis among study participants

Three prominent amino acid patterns were identified using PCA, collectively accounting for 97.6% of the total variance in amino acid intake. Table 1 presents the factor loadings and the variances of each pattern and Fig. 1 depicts the scree plot exhibiting the eigenvalues. Pattern 1 exhibited higher loads of arginine, glycine, tryptophan, aspartic acids, cysteine and alanine; pattern 2 was characterized by higher loads of proline, phenylalanine, valine, serine, glutamic acid, tyrosine and leucine, whereas pattern 3 displayed higher loads of histidine, lysine, methionine, isoleucine and threonine.

**Table 1:** Component loadings for dietary amino acid patterns

	**Amino acid patterns**		
	Pattern 1	Pattern 2	Pattern 3
**Arginine**	0.899	-	-
**Glycine**	0.814	-	-
**Tryptophan**	0.651	-	-
**Aspartic acid**	0.571	-	-
**Cysteine**	0.524	-	-
**Alanine**	0.455	-	-
**Proline**	-	0.891	-
**Phenylalanine**	-	0.699	-
**Valine**	-	0.675	-
**Serine**	-	0.656	-
**Glutamine**	-	0.520	-
**Tyrosine**	-	0.501	-
**Leucine**	-	0.495	-
**Histidine**	-	-	0.763
**Lysine**	-	-	0.694
**Methionine**	-	-	0.657
**Isoleucine**	-	-	0.433
**Threonine**	-	-	0.409
**Variance**	94.092	2.092	1.478

Factor loadings of food patterns measured by factor analysis. Absolute value > 0.4

Table 2 provides an overview of the demographic, anthropometric, and biochemical characteristics of the study participants across the tertiles of dietary amino acid patterns. Participants in upper tertiles of pattern 1 showed higher WC and WHR (*P* = 0.01 and *P* = 0.04, respectively). Moreover, individuals in the upper tertiles of pattern 2 were more likely to be male (*P* = 0.03) and had higher WC (*P* = 0.03) and FFM (*P* = 0.007). Individuals in the higher tertiles of pattern 3 exhibited higher serum TG levels (*P* = 0.01) compared to those in the lower tertiles. Additionally, participants in the upper tertiles of all three amino acid patterns had significantly higher energy intake and appetite (*P* < 0.001 for energy intake and *P* = 0.02 for appetite across all patterns).

**Table 2:** General demographic characteristics of study participants by tertiles of amino acid patterns

**Variable**	**Pattern 1**				**Pattern 2**				**Pattern 3**			
	T1 *n* = 113	T2 *n* = 112	T3 *n* = 110	***P* Value**	T1 *n* = 112	T2 *n* = 110	T3 *n* = 113	***P* Value**	T1 *n* = 109	T2 *n* = 114	T3 *n* = 112	***P* Value**
Age (y) Median (IQR)	40.00 (11.00)	36.00 (10.75)	39.00 (12.75)	0.152	40.00 (11.00)	37.00 (11.00)	38.00 (13.00)	0.783	39.00 (10.75)	38.00 (11.00)	37.50 (12.75)	0.692
Sex (Male) n (%)	57 (50.4%)	69 (61.6%)	68 (60.2%)	0.140	56 (80.0%)	66 (58.4%)	72 (63.7%)	**0.038**	56 (50.0%)	70 (61.4%)	68 (60.7%)	0.105
Education (diploma ≤) n (%)	54 (93.1%)	59 (95.2%)	61 (89.5%)	0.880	56 (91.8%)	61 (92.3%)	57 (93.4%)	0.421	69 (94.5%)	48 (87.3%)	56 (94.9%)	0.169
Marital status (Single) n (%)	14 (12.4%)	17 (15.2%)	17 (15.0%)	0.384	16 (14.0%)	13 (11.5%)	19 (14.2%)	0.743	14 (12.5%)	13 (11.4%)	18 (16.1%)	0.382
Family size (> 4) n (%)	9 (15.5%)	7 (11.3%)	11 (16.2%)	0.545	9 (14.8%)	8 (12.2%)	10 (16.1%)	0.967	11 (15.1%)	7 (12.4%)	9 (15.1%)	0.981
BMI (kg/m^2^) Median (IQR)	33.34 (5.75)	34.12 (5.03)	34.58 (5.18)	0.055	33.19 (5.59)	34.19 (5.36)	34.37 (4.67)	0.315	33.75 (5.36)	35.04 (5.81)	33.57 (3.68)	0.626
WC (cm) Mean (SD)	104.83 (8.77)	106.59 (10.40)	108.68 (9.46)	**0.011**	104.84 (8.66)	107.19 (9.95)	108.06 (10.10)	**0.035**	106.05 (9.83)	106.60 (9.78)	107.46 (9.40)	0.545
WHR Mean (SD)	0.92 (0.08)	0.92 (0.07)	0.94 (0.06)	**0.043**	0.92 (0.07)	0.93 (0.08)	0.94 (0.06)	0.190	0.92 (0.08)	0.93 (0.07)	0.93 (0.07)	0.383
FM (%) Median (IQR)	34.90 (10.45)	32.40 (12.43)	32.85 (14.05)	0.942	33.80 (10.70)	32.30 (12.80)	32.90 (14.30)	0.966	34.00 (11.28)	35.20 (16.00)	30.40 (12.90)	0.469
FFM (%) Median (IQR)	55.00 (22.10)	64.05 (22.75)	65.30 (24.52)	0.065	52.90 (20.80)	63.10 (21.90)	66.50 (23.90)	**0.007**	58.15 (22.80)	65.20 (21.60)	64.40 (24.63)	0.313
SES Score Mean (SD)	9.51 (2.69)	10.24 (2.46)	10.08 (2.37)	0.252	9.47 (2.74)	10.15 (2.44)	10.24 (2.29)	0.179	9.50 (2.68)	10.16 (2.65)	10.33 (2.05)	0.130
Energy (Kcal) Median (IQR)	2078.28 (597.18)	2960.31 (877.05)	3477.74 (1003.59)	**< 0.001**	2121.42 (786.98)	2945.59 (870.75)	3485.90 (1319.24)	**< 0.001**	2293.44 (643.34)	3084.18 (862.00)	3516.12 (1229.23)	**< 0.001**
Appetite Median (IQR)	31.07 (10.50)	35.00 (8.75)	33.55 (12.75)	**0.022**	32.00 (10.00)	34.00 (13.00)	35.00 (12.00)	**0.032**	31.82 (9.22)	33.00 (10.00)	36.00 (13.75)	**0.027**
LDL (mg/dl) Median (IQR)	120.60 (36.00)	122.20 (34.95)	111.40 (47.35)	0.112	120.60 (31.60)	122.00 (45.20)	110.00 (41.40)	0.154	121.00 (32.15)	120.20 (49.30)	111.40 (40.75)	0.411
HDL (mg/dl) Median (IQR)	45.00 (14.00)	43.50 (10.50)	45.00 (13.00)	0.445	45.00 (16.00)	42.00 (10.00)	45.00 (13.00)	0.243	45.00 (13.00)	42.00 (16.00)	45.00 (13.55)	0.515
TG (mg/dl) Median (IQR)	105.00 (50.00)	103.50 (62.25)	118.50 (82.25)	0.503	98.00 (48.0)	120.00 (64.00)	111.00 (77.00)	0.453	100.00 (45.75)	111.00 (80.50)	118.00 (72.00)	**0.011**
TC (mg/dl) Mean (SD)	196.80 (39.81)	190.66 (36.30)	187.83 (33.66)	0.292	195.35 (38.42)	193.22 (39.15)	186.78 (32.12)	0.241	192.97 (36.81)	192.36 (39.82)	189.96 (33.62)	0.977
FBS (mg/dl) Median (IQR)	90.00 (14.00)	91.00 (11.75)	92.00 (20.25)	0.380	90.00 (13.00)	91.00 (15.00)	92.00 (15.00)	0.936	88.00 (13.00)	93.00 (16.00)	90.50 (13.25)	0.682
Insulin (mIU/l) Median (IQR)	12.60 (11.00)	13.10 (15.33)	13.90 (16.50)	0.714	13.00 (10.00)	13.80 (16.40)	13.20 (15.20)	0.695	12.40 (11.80)	13.40 (13.75)	13.25 (17.75)	0.665
SBP (mmHg) Median (IQR)	110.00 (19.00)	110.00 (15.00)	117.50 (23.75)	0.872	110.00 (23.00)	110.00 (15.00)	115.00 (18.00)	0.954	121.40 (15.63)	110.00 (20.00)	111.00 (10.00)	0.161
DBP (mmHg) Median (IQR)	80.00 (11.50)	70.00 (10.00)	75.00 (16.00)	0.270	75.00 (10.00)	75.00 (15.00)	70.00 (10.00)	0.831	81.66 (10.96)	75.00 (15.00)	70.00 (10.00)	0.773

Continuous values are shown as median (interquartile range) or mean (standard deviation). Categorical values are shown as number (%); Kruskal-Wallis test was used for estimate *p*-value of non-parametric variables. One-way ANOVA test was used for estimate *p*-value of parametric variables

*BMI* Body mass index, *WC* Waist Circumference, *WHR* Waist-to-hip ratio, *FM* Fat Mass, *FFM* Fat Free Mass, *SES* Socio-economic status, *BMR* Basal Metabolic Rate, *LDL-C* Low Density Lipoprotein Cholesterol, *HDL-C* High Density Lipoprotein Cholesterol, *TG* Triglyceride, *TC* Total Cholesterol, *SBP* Systolic Blood Pressure, *DBP* Diastolic Blood Pressure

Table 3 displays the dietary energy and nutrient intakes of individuals across different tertiles of dietary amino acid patterns. Those in the upper tertiles had significantly higher intake of energy, protein, total cholesterol (Cho), saturated fat (SF), monounsaturated fatty acids (MUFA), polyunsaturated fatty acids (PUFA) and fiber (*P* < 0.01). In pattern 1 and pattern 3, subjects in the higher tertiles had lower intakes of carbohydrates (*P* < 0.01).

**Table 3:** Dietary energy and nutrient intake of participants according to amino acid pattern

**Variable**	**Pattern 1**				**Pattern 2**				**Pattern 3**			
	T1	T2	T3	*P* Value*	T1	T2	T3	*P* Value *	T1	T2	T3	*P* Value *
	**Mean (SD)**	**Mean (SD)**	**Mean (SD)**		**Mean (SD)**	**Mean (SD)**	**Mean (SD)**		**Mean (SD)**	**Mean (SD)**	**Mean (SD)**	
**Energy(kcal/d)**	2163.83 (550.84)	3069.71 (837.16)	3817.16 (1117.36)	**< 0.001**	2183.45 (585.17)	2990.93 (792.39)	3860.81 (1118.74)	**< 0.001**	2293.44 (643.34)	3043.76 (952.19)	3706.77 (1147.89)	**< 0.001**
**CHO (% of energy)**	61.28 (7.52)	59.76 (7.78)	59.03 (6.72)	**< 0.001**	60.69 (7.71)	60.17 (7.72)	59.22 (6.70)	0.325	61.58 (7.90)	60.17 (7.66)	58.33 (6.21)	**0.004**
**Pro (% of energy)**	12.94 (2.06)	13.05 (2.08)	14.11 (2.21)	**< 0.001**	12.94 (2.30)	13.14 (1.99)	14.03 (2.10)	**< 0.001**	12.42 (1.81)	13.21 (1.96)	14.47 (2.25)	**< 0.001**
**Fat (%of energy)**	28.70 (6.72)	30.33 (7.36)	29.92 (6.20)	0.175	29.20 (7.26)	29.99 (7.10)	29.74 (5.99)	0.678	28.98 (7.44)	29.93 (6.94)	30.02 (5.92)	0.450
**Cholesterol (g)**	213.83 (239.14)	289.69 (133.80)	388.32 (171.94)	**< 0.001**	212.92 (234.44)	293.66 (153.39)	383.89 (166.19)	**< 0.001**	209.92 (241.59)	288.67 (142.57)	392.72 (159.51)	**< 0.001**
**SF (g)**	20.00 (7.31)	30.56 (15.74)	37.44 (14.95)	**< 0.001**	19.92 (7.33)	28.60 (13.78)	39.30 (15.64)	**< 0.001**	20.16 (7.23)	29.24 (14.02)	38.50 (16.16)	**< 0.001**
**MUFA (g)**	22.50 (7.59)	35.29 (17.52)	42.06 (16.82)	**< 0.001**	24.12 (11.51)	34.10 (16.29)	41.44 (17.16)	**< 0.001**	24.37 (10.72)	34.63 (18.08)	40.72 (16.27)	**< 0.001**
**PUFA (g)**	15.26 (6.12)	24.08 (14.18)	28.59 (14.15)	**< 0.001**	16.84 (9.94)	22.63 (11.23)	28.34 (15.46)	**< 0.001**	17.60 (9.48)	23.30 (14.18)	26.95 (13.94)	**< 0.001**
**Fiber(g)**	55.71 (36.22)	69.81 (35.46)	88.31 (51.35)	**< 0.001**	54.67 (31.07)	70.35 (36.17)	91.24 (54.14)	**< 0.001**	62.50 (36.41)	69.60 (47.04)	86.23 (46.56)	**0.007**

All data are mean (± SD)

*CHO* Carbohydrate, *Pro* Protein, *SF* Saturated Fat, *MUFA* Monounsaturated Fatty Acid, *PUFA* Polyunsaturated Fatty Acid

P* values derived from unadjusted ANOVA

Table 4 presents the ORs and 95% CIs for participants’ anthropometric variables based on the tertiles of dietary amino acid patterns. Participants in the highest tertiles of pattern 1 exhibited a higher BMI in the crude model (OR = 1.06, CI = 1.00–1.12, *P* = 0.03). increased adherence to pattern 1 and pattern 2 was associated with higher WC in the crude model (OR = 1.04, CI = 1.01–1.07, *P* = 0.003 and OR = 1.03, CI = 1.00–1.05, *P* = 0.01, respectively). However, after adjusting for potential confounders, the significance of the association between BMI and WC and amino acid patterns was no longer observed. The third tertile of pattern 2 was consistently associated with higher FFM across all three models, in comparison to the first tertiles (OR = 1.04, CI = 1.01–1.08, *P* = 0.002 for the crud model, OR = 1.08, CI = 1.01–1.16, *P* = 0.01 for model II and OR = 1.10, CI = 1.00–1.20, *P* = 0.03, for model III).

**Table 4:** Association between anthropometric variables and amino acid patterns among participants

**Variable**	**Tertiles of Amino Acid Pattern 1**					**Tertiles of Amino Acid Pattern 2**					**Tertiles of Amino Acid Pattern 3**				
	T_1_	T2		T3		T_1_	T2		T3		T_1_	T2		T3	
		OR (CI)	*P*-value	OR (CI)	*P*-value		OR (CI)	*P*-value	OR (CI)	*P*-value		OR (CI)	*P*-value	OR (CI)	*P*-value
**BMI (kg/m2)**															
Model I	**1 REF**	1.021 (0.966–1.080)	0.456	1.062 (1.006–1.123)	**0.031**	**1 REF**	1.043 (0.987–1.102)	0.137	1.039 (0.983–1.098)	0.176	**1 REF**	0.990 (0.938–1.045)	0.714	1.004 (0.951–1.059)	0.891
Model II		1.032 (0.973–1.094)	0.297	1.067 (0.999–1.122)	0.069		1.056 (0.997–1.118)	0.065	1.059 (0.999–1.118)	0.054		1.003 (0.949–1.061)	0.905	1.016 (0.960–1.074)	0.589
Model III		1.014 (0.944–1.089)	0.699	1.063 (0.982–1.151)	0.129		1.049 (0.979–1.124)	0.176	1.044 (0.964–1.131)	0.287		0.978 (0.918–1.041)	0.481	0.979 (0.914–1.049)	0.555
**WC (cm)**															
Model I	**1 REF**	1.020 (0.992–1.050)	0.159	1.043 (1.014–1.073)	**0.003**	**1 REF**	1.027 (0.998–1.056)	0.064	1.036 (1.008–1.056)	**0.013**	**1 REF**	1.006 (0.979–1.034)	0.667	0.988 (1.043–1.015)	0.274
Model II		1.016 (0.986–1.046)	0.309	1.030 (0.998–1.059)	0.114		1.023 (0.994–1.050)	0.122	1.031 (1.001–1.062)	0.079		0.998 (0.970–1.027)	0.901	0.680 (0.390–1.185)	0.511
Model III		1.007 (0.971–1.046)	0.696	1.033 (0.991–1.076)	0.123		1.020 (0.983–1.057)	0.296	1.022 (0.981–1.065)	0.298		0.985 (0.953–1.017)	0.348	0.990 (0.956–1.026)	0.591
**WHR (cm)**															
Model I	**1 REF**	1.017 (0.986–1.049)	0.282	1.016 (0.985–1.048)	0.309	**1 REF**	0.996 (0.699–1.027)	0.804	1.016 (0.986–1.047)	0.304	**1 REF**	0.984 (0.955–1.015)	0.310	0.992 (0.963–1.022)	0.619
Model II		1.025 (0.992–1.059)	0.137	1.024 (0.991–1.058)	0.150		1.002 (0.970–1.035)	0.926	1.026 (0.994–1.060)	0.108		0.990 (0.959–1.021)	0.517	0.999 (0.968–1.030)	0.939
Model III		1.025 (0.981–1.070)	0.273	1.023 (0.985–1.063)	0.263		1.004 (0.967–1.042)	0.832	1.032 (0.988–1.078)	0.157		0.984 (0.951–1.019)	0.372	0.989 (0.953–1.027)	0.560
**FM (%)**															
Model I	**1 REF**	1.012 (0.973–1.053)	0.556	1.006 (0.967–1.046)	0.779	**1 REF**	1.008 (0.970–1.048)	0.683	1.011 (0.972–1.051)	0.584	**1 REF**	1.014 (0.977–1.053)	0.469	0.993 (0.956–1.033)	0.735
Model II		1.039 (0.988–1.094)	0.138	1.034 (0.983–1.088)	0.192		1.035 (0.983–1.089)	0.192	1.052 (1.000–1.107)	0.052		1.028 (0.982–1.076)	0.243	1.009 (0.962–1.058)	0.714
Model III		1.032 (0.971–1.097)	0.311	1.030 (0.963–1.103)	0.390		1.037 (0.977–1.101)	0.228	1.067 (0.997–1.142)	0.062		1.019 (0.969–1.071)	0.464	0.999 (0.944–1.057)	0.976
**FFM (%)**															
Model I	**1 REF**	1.019 (0.989–1.050)	0.207	1.025 (0.995–1.056)	0.081	**1 REF**	1.031 (1.001–1.062)	**0.044**	1.048 (1.017–1.080)	**0.002**	**1 REF**	1.016 (0.987–1.046)	0.276	1.016 (0.988–1.045)	0.253
Model II		1.038 (0.972–1.109)	0.264	1.063 (0.996–1.134)	0.066		1.079 (1.009–1.153)	**0.025**	1.088 (1.017–1.164)	**0.014**		1.048 (0.957–1.053)	0.150	1.008 (0.948–1.072)	0.804
Model III		1.036 (0.958–1.120)	0.373	1.085 (0.993–1.185)	0.070		1.091 (1.012–1.177)	**0.022**	1.102 (1.000–1.206)	**0.038**		1.039 (0.970–1.113)	0.273	1.005 (0.934–1.081)	0.893

The multivariate multinomial logistic regression was used for estimation of ORs and 95% CI. Model I: crude, Model II: adjusted for age and sex, Model III: adjusted for age, sex and energy intake

*BMI* Body mass index, *WC* Waist Circumference, *WHR* Waist-to-hip ratio, *FM* Fat Mass, *FFM* Fat Free Mass, *OR* Odds ratio, *CI* Confidence interval

The ORs and 95% CIs for biochemical variables of the subjects displayed in Table 5. Individuals in the third tertiles of pattern 1 and pattern 2, were more likely to have lower level of LDL (OR = 0.99, CI = 0.98–0.99, *P* = 0.02 for both pattern in the crude model, and OR = 0.99, CI = 0.98–0.99, *P* = 0.01 for both pattern in model I) compared to those in the first tertiles, both in the crude and age- and sex-adjusted models. In model II, pattern 2 showed a significant decrease in TC level in the third tertiles (OR = 0.99, CI = 0.98–0.99, *P* = 0.04). Furthermore, the results indicated a positive association between the third amino acid pattern and increased TG levels, even after controlling for potential confounders such as age, sex, BMI, PA and energy intake (OR = 1.004, CI = 1.000–1.007, *P* = 0.03).

**Table 5:** Association between biochemical variables and amino acid patterns among participants

**Variable**	**Tertiles of Amino Acid Pattern 1**					**Tertiles of Amino Acid Pattern 2**					**Tertiles of Amino Acid Pattern 3**				
	T_1_	T2		T3		T_1_	T2		T3		T_1_	T2		T3	
		OR (CI)	*P*-value	OR (CI)	*P*-value		OR (CI)	*P*-value	OR (CI)	*P*-value		OR (CI)	*P*-value	OR (CI)	*P*-value
**LDL (mg/dl)**															
Model I	**1 REF**	0.997 (0.989–1.005)	0.442	0.990 (0.982–0.999)	**0.023**	**1 REF**	0.995 (0.987–1.003)	0.243	0.990 (0.982–0.999)	**0.024**	**1 REF**	1.001 (0.992–1.009)	0.890	0.995 (0.987–1.003)	0.233
Model II		0.997 (0.989–1.006)	0.517	0.990 (0.982–0.998)	**0.019**		0.995 (0.987–1.003)	0.232	0.990 (0.981–0.998)	**0.019**		1.000 (0.992–1.008)	0.963	0.995 (0.989–1.003)	0.218
Model III		1.003 (0.989–1.017)	0.654	0.989 (0.972–1.006)	0.202		1.001 (0.988–1.015)	0.827	0.988 (0.971–1.005)	0.176		0.999 (0.986–1.012)	0.876	0.991 (0.977–1.006)	0.224
**HDL (mg/dl)**															
Model I	**1 REF**	0.983 (0.956–1.010)	0.213	0.986 (0.960–1.014)	0.329	**1 REF**	0.981 (0.954–1.008)	0.166	0.988 (0.961–1.015)	0.379	**1 REF**	0.997 (0.970–1.024)	0.803	0.985 (0.958–1.013)	0.287
Model II		0.989 (0.961–1.018)	0.457	0.992 (0.964–1.021)	0.576		0.985 (0.957–1.014)	0.302	0.996 (0.968–1.025)	0.792		1.005 (0.976–1.034)	0.757	0.991 (0.962–1.020)	0.543
Model III		0.980 (0.929–1.033)	0.451	0.997 (0.940–1.058)	0.919		0.975 (0.928–1.024)	0.313	1.004 (0.947–1.065)	0.890		0.987 (0.941–1.035)	0.589	0.996 (0.946–1.048)	0.867
**TG (mg/dl)**															
Model I	**1 REF**	1.000 (0.997–0.003)	0.992	1.000 (0.997–1.003)	0.941	**1 REF**	1.002 (0.999–1.005)	0.271	1.001 (0.998–1.004)	0.420	**1 REF**	1.004 (1.000–1.007)	**0.027**	1.004 (1.001–1.007)	**0.021**
Model II		1.000 (0.997–1.003)	0.847	0.999 (0.997–1.002)	0.989		1.001 (0.998–1.004)	0.397	1.001 (0.998–1.004)	0.712		1.004 (1.001–1.008)	**0.046**	1.004 (1.000–1.007)	**0.040**
Model III		0.998 (0.989–1.006)	0.581	1.001 (0.992–1.010)	0.852		1.006 (0.998–1.014)	0.126	1.003 (0.994–1.013)	0.505		1.006 (0.999–1.014)	0.097	1.004 (1.000–1.007)	**0.032**
**TC (mg/dl)**															
Model I	**1 REF**	0.995 (0.988–1.003)	0.212	0.993 (0.986–1.000)	0.068	**1 REF**	0.998 (0.991–1.006)	0.664	0.994 (0.986–1.001)	0.081	**1 REF**	1.000 (0.992–1.007)	0.901	0.998 (0.991–1.005)	0.542
Model II		0.996 (0.989–1.003)	0.266	0.993 (0.986–1.000)	**0.057**		0.998 (0.991–1.005)	0.630	0.992 (0.985–0.999)	**0.048**		0.999 (0.992–1.006)	0.783	0.998 (0.990–1.005)	0.515
Model III		1.000 (0.987–1.013)	0.981	0.991 (0.976–1.007)	0.274		1.003 (0.991–1.015)	0.654	0.992 (0.977–1.008)	0.321		1.002 (0.990–1.014)	0.797	0.995 (0.982–1.008)	0.476
**FBS (mg/dl)**															
Model I	**1 REF**	0.990 (0.972–1.009)	0.310	1.012 (0.997–1.028)	0.128	**1 REF**	1.009 (0.994–1.024)	0.262	1.006 (0.990–1.022)	0.467	**1 REF**	1.011 (0.994–1.028)	0.218	1.013 (0.997–1.030)	0.117
Model II		0.992 (0.973–1.011)	0.400	1.011 (0.996–1.027)	0.155		1.009 (0.993–1.025)	0.284	1.005 (0.989–1.021)	0.542		1.010 (0.992–1.028)	0.269	1.014 (0.996–1.031)	0.126
Model III		1.000 (0.964–1.037)	0.979	1.016 (0.979–1.054)	0.410		1.029 (0.994–1.067)	0.109	1.023 (0.986–1.062)	0.232		1.030 (0.997–1.063)	0.077	1.028 (0.994–1.062)	0.104
**Insulin (mIU/l)**															
Model I	**1 REF**	0.984 (0.955–1.014)	0.984	1.008 (0.986–1.030)	0.493	**1 REF**	1.007 (0.982–1.033)	0.592	1.010 (0.985–1.036)	0.431	**1 REF**	1.002 (0.976–1.030)	0.865	1.014 (0.989–1.039)	0.273
Model II		0.985 (0.956–1.015)	0.337	1.007 (0.984–1.031)	0.543		1.009 (0.981–1.037)	0.536	1.011 (0.984–1.039)	0.421		1.003 (0.976–1.032)	0.816	1.014 (0.988–1.041)	0.282
Model III		1.022 (0.971–1.075)	0.405	1.033 (0.975–1.095)	0.268		1.021 (0.974–1.070)	0.395	1.012 (0.953–1.075)	0.693		0.995 (0.948–1.044)	0.839	1.041 (0.992–1.094)	0.104
**SBP (mmHg)**															
Model I	**1 REF**	1.003 (0.987–1.019)	0.726	0.993 (0.977–1.009)	0.399	**1 REF**	1.000 (0.984–1.016)	0.977	0.995 (0.979–1.011)	0.541	**1 REF**	1.015 (0.998–1.032)	0.080	1.000 (0.984–1.016)	0.984
Model II		1.005 (0.987–1.023)	0.603	0.989 (0.972–1.007)	0.229		0.997 (0.980–1.015)	0.776	0.990 (0.973–1.007)	0.252		1.012 (0.994–1.030)	0.197	0.998 (0.981–1.015)	0.828
Model III		1.023 (0.986–1.061)	0.220	1.017 (0.976–1.059)	0.421		0.998 (0.967–1.031)	0.919	0.988 (0.949–1.029)	0.557		1.007 (0.976–1.039)	0.653	0.999 (0.964–1.035)	0.956
**DBP (mmHg)**															
Model I	**1 REF**	0.987 (0.964–1.009)	0.244	0.986 (0.963–1.008)	0.211	**1 REF**	0.999 (0.977–1.022)	0.953	0.989 (0.967–1.012)	0.341	**1 REF**	1.007 (0.984–1.030)	0.549	0.992 (0.970–1.015)	0.494
Model II		0.988 (0.964–1.012)	0.327	0.981 (0.958–1.005)	0.128		0.998 (0.974–1.022)	0.842	0.984 (0.961–1.008)	0.189		1.002 (0.979–1.027)	0.841	0.990 (0.967–1.014)	0.400
Model III		0.995 (0.952–1.040)	0.814	1.005 (0.957–1.054)	0.853		1.018 (0.978–1.060)	0.375	0.992 (0.945–1.042)	0.756		0.988 (0.951–1.027)	0.555	0.977 (0.935–1.020)	0.282

The multivariate multinomial logistic regression was used for estimation of ORs and 95% CI. Model I: crude, Model II: adjusted for age and sex, Model III: adjusted for age, sex, BMI and energy intake

*LDL-C* Low Density Lipoprotein Cholesterol, *HDL-C* High Density Lipoprotein Cholesterol, *TG* Triglyceride, *TC* Total Cholesterol, *FBS* Fasting Blood Sugar, *SBP* Systolic Blood Pressure, *DBP* Diastolic Blood Pressure, *OR* Odds ratio, *CI* Confidence interval

## Discussion

The study revealed that factors 1 and 2, which had a major contribution of amino acids such as arginine, glycine, tryptophan, aspartic acid for factor 1, and proline, phenylalanine, valine, serine, and glutamic acid for factor 2, exhibited a negative correlation with serum LDL and total cholesterol levels. Conversely, factor 3, which consisted of histidine, lysine, methionine, and isoleucine, displayed an association with elevated serum triglycerides.

Among the amino acids contributing to a more favorable lipid profile (Factors 1 and 2), arginine -the highest loading component in Factor 1 - has undergone extensive investigation. Available evidence suggests that arginine, as a substrate for nitric oxide (NO) synthesis, fosters lipid oxidation by increasing the phosphorylation of hormone-sensitive lipase [31]. The L-arginine-nitric oxide pathway, through which cell signal protein can be activated, has shown to regulate carbohydrate and lipid metabolism [32]. Furthermore, the functional relevance of L- arginine in T2DM has been shown to improve insulin sensitivity, glucose disposal and reduce glucose intolerance [33, 34]. Arginine provides the substrate to improve endothelial function and diminish the susceptibility of LDL to oxidation in patients with stable coronary artery disease, exerting its effects through multiple mechanisms, including the direct, NO-dependent, and NO-independent antioxidant capacity [35]. Additionally, in vivo supplementation of L-arginine has shown a reduction in oxidative stress by restoring superoxide dismutase and catalase activities as well as glutathione concentration [36]. Steven et al. conducted a study investigating the effects of plant extracts on blood parameters and oxidative/antioxidant status and concluded that plant-derived L-arginine improved lipid and glycemic profiles, reduced oxidant parameters, increased antioxidant tissue activity [37]. While dietary glutamate has shown an inverse association with cardiometabolic risk, the impact of glutamine on cardiometabolic risk appears to be unclear [38, 39]. However, it is important to state that our data did not detect these two amino acids. Other amino acids found in the protective factors have also been studied for their antioxidative effects on cardiometabolic factors. Glutathione (GSH), a major endogenous antioxidants synthesized in almost all tissues by sequential addition of the precursor amino acids cysteine, glutamic acid and glycine, appears to be associated with cardiometabolic risk due to its antioxidant role [38–41]. In addition to these amino acids, other studies have observed an increase in the total antioxidant capacity of plasma in connection with other antioxidant amino acids, including tryptophan and tyrosine (predominant in Factor 1 and 2, respectively) [42] which our conclusion is consistent with these findings. Rom et al. also found an anti-atherogenic role of glycine, cysteine, alanine, and leucine, which decreased macrophage triglyceride content by reducing VLDL uptake [22]. Furthermore, F Teymoori et al. demonstrated a negative association between higher intake of tryptophan and changes in serum TG, LDL-C, and TC levels which could be explained by the role of tryptophan as a serotonin precursor [43]. Serotonin, which assists in regulating glucose and lipid metabolism, has emerged as a novel therapeutic target for lipid metabolic disorders [44]. Phenylalanine and tyrosine, both precursors to the neurotransmitter dopamine, which plays a role in appetite and mood regulating, have been negatively associated with increased food intake [45]. As a non-essential amino acid, proline possesses antioxidant and anti-inflammatory effects in animal models, which may contribute to its beneficial effects on lipid metabolism and cardiovascular health [46]. High intake of sulfur-containing AAs, including methionine and cysteine, may contribute to cardiometabolic risk factors such as higher TCs and TGs by increasing oxidative stress and insulin resistance [43, 47]. Our results are in line with these findings for methionine, while cysteine in our study appears to have a cardiometabolic protective role which can be explained through modulation of redox status. Methionine, as a precursor of homocysteine, may play an increasing role in cardiovascular risk [48]. Nevertheless, evidence for the effect of methionine in interaction with other amino acids and the protein source is limited [49]. In an animal model, methionine supplementation result in hypercholesterolemia [50]. Moreover, a positive relationship between homocysteine and liver triglyceride levels has been demonstrated [51]. A potential cardioprotective role has been demonstrated for the intake of 7 amino acids (arginine, cysteine, glutamic acid, glycine, histidine, leucine, and tyrosine) [14], which is consistent with our results except for histidine. Leucine and valine, on the other hand, play a role in regulating lipid metabolism and insulin sensitivity by activating the mammalian target of rapamycin (mTOR) signaling pathway [46, 52].

**Fig. 2:**
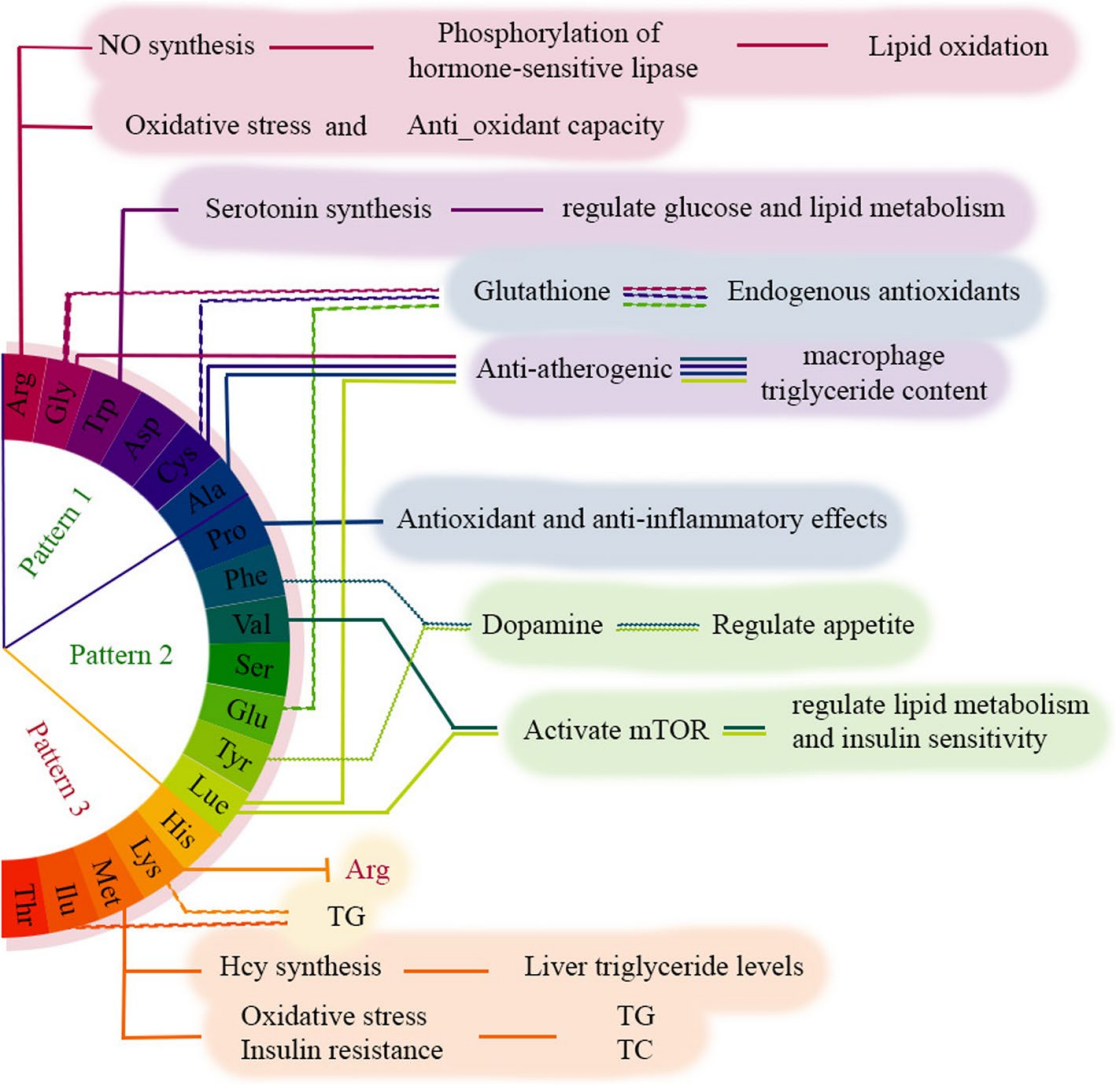
Mechanistic pathways of amino acids role in cardio-metabolic factors. Arg contributes in cardio metabolic health by increasing lipid oxidation through No synthesis as well as its anti-oxidant role. Serotonin which derived from Trp has a therapeutic role in regulating glucose and fat metabolism. Gly, Cys and Glu are involved in the synthesis of glutathione - an endogenous antioxidant - which has a cardioprotective role. Amino acids such as Gly, Cys, Ala and Lue had anti-atherogenic role through reducing VLDL uptake and macrophage triglyceride content. Phe and Tyr regulate appetite by dopamine synthesis. Val and Lue contribute in regulating lipid metabolism and insulin sensitivity by activating mTOR. Met—a sulfur-containing amino acid—increases oxidative stress and insulin resistance, as well as liver triglyceride levels. It has been shown a positive association between Lys and Ilu and serum TG level. Abbreviates: Arg, arginine; Gly, glycine; Trp, tryptophan; Asp, aspartate; Cys, cysteine; Ala, alanine; Pro, Proline; Phe, phenylalanine; Val, valine Ser, serine; Glu, glutamate; Tyr, tyrosine; Lue, Lucine; His, histidine; Lys, lysine; Met, methionine; Ilu, isoleucine; Thr, threonine; NO, nitric oxide; TG, triglyceride; Hcy, homocysteine; TC, total cholesterol. Pattern 1: Arg, Gly, Trp, Asp, Cys and Ala, Pattern 2: Pro, Phe, Val, Ser, Glu, Tyr and Lue, pattern 3: His, Lys, Met, Ilu and Thr

In contrast to our results, animal studies have shown that histidine supplementation reduce liver TGs and TCs content [53]. Additionally, studies by Javidan et al. reported a positive correlation between dietary lysine and isoleucine and serum TG levels, contradicting the results of Bel-Serrat et al. [54, 55]. Unfavorable effects of lysine on hypertriglyceridemia and hypercholesterolemia have also been reported in animal studies [56, 57]. It appears that the specific composition of accompanying amino acid influences the role of dietary amino acids in the body. In animal proteins, lysine competes with the intracellular transport of arginine due to a higher lysine-to-arginine ratio, thereby altering the physiological effects of arginine in the body [58, 59]. A randomized controlled crossover trial has shown that a low lysine-to-arginine ratio diet reduces postprandial TG levels [60]. Therefore, there may be substances associated with food proteins that strongly affect the function of amino acids. Figure 2 illustrates some of the mechanistic pathways depicting the involvement of amino acids in cardiometabolic factors.

To the best of our knowledge, this is the first study to examine the relationship between dietary amino acid patterns and cardiometabolic risk among Iranian adults. All phases of recruitment and data collection were conducted by a trained nutritionist, increasing the accuracy of the evaluation. Additionally, to achieve an independent relationship between dietary amino acid patterns and cardiometabolic factors, numerous potential confounders were considered in the analysis. The use of factor analysis to identify amino acid patterns not only better describes the dietary amino acid composition in our population but also takes into account any inter-correlation between amino acids and their cumulative effects. However, our study does have some potential limitations; First, the cross-sectional design complicates causal inference and longitudinal studies are required to better understand the cause-effect relationship. Second, the study had a small number of participants. Third, despite adjusting for the confounders, residual confounding factors may still exist. Forth, the FFQ used in this study was not originally developed for assessing amino acid intake. FFQs have the advantage of assessing participants’ usual dietary intake over a long period of time, but the risk of recall and data reporting errors is greater. However, the FFQ is the most widely used and population-based questionnaire for dietary assessment, which has been successfully validated for the Iranian population [28].

## Conclusion

This study found that two identified amino acid patterns, factor 1 and factor 2, were negatively associated with serum LDL and TC levels. These patterns had major contributions from amino acids such as arginine, glycine, tryptophan, aspartic acid, proline, phenylalanine, valine, serine, and glutamic acid. On the other hand, factor 3, consisting of histidine, lysine, methionine, and isoleucine, was associated with higher serum TG levels. These findings highlight the importance of specific amino acid patterns in managing cardiometabolic risk factors in individuals with obesity.

## Data Availability

The datasets generated and/or analyzed during the current study are not publicly available due to some restrictions that applied by the ethical committee but are available from the corresponding author on reasonable request.
